# (9*H*-Carbazol-9-ylmeth­yl)diethyl­amine

**DOI:** 10.1107/S1600536810047197

**Published:** 2010-11-20

**Authors:** Wei-Jin Gu, Zi-Ting Lin, Bing-Xiang Wang

**Affiliations:** a Department of Applied Chemistry, Nanjing Normal University, Nanjing 210097, People’s Republic of China

## Abstract

The asymmetric unit of the title compound, C_17_H_20_N_2_, contains two mol­ecules, whose bond lengths and angles differ only slightly. In the crystal, neighbouring mol­ecules form pillar structures *via* edge-to-face π–π stacking inter­actions [edge-to-face distances = 3.538 (3) and 3.496 (3)Å].

## Related literature

Carbazole-based compounds are widely used in OLEDs as emitters because of their intense luminescence, see: Adhikari *et al.* (2007 ▶); Liu *et al.* (2006 ▶); Palayangoda *et al.* (2008 ▶) and as organic fluorescence probes, see: Hao *et al.* (2010 ▶); Pappayee & Mishra, (2000 ▶). For our studies of organic fluorescence probes, see: Shen *et al.* (2006 ▶, 2008 ▶). For the preparation of the title compound, see: Gu *et al.* (1997 ▶).
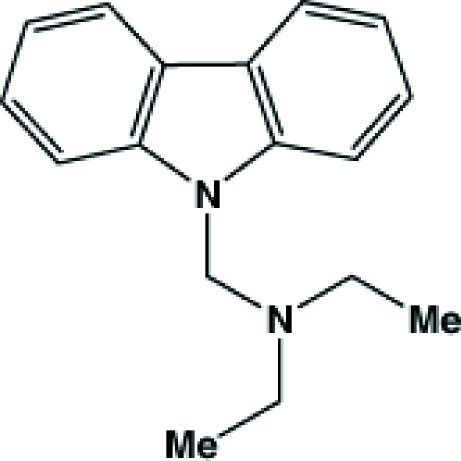


## Experimental

### 

#### Crystal data

C_17_H_20_N_2_
*M* *_r_* = 252.35
Monoclinic, 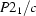

*a* = 24.338 (2) Å
*b* = 6.3216 (11) Å
*c* = 19.133 (2) Å
β = 104.109 (2)°
*V* = 2854.9 (6) Å^3^
*Z* = 8
Mo *K*α radiation
μ = 0.07 mm^−1^
*T* = 291 K
0.28 × 0.24 × 0.22 mm

#### Data collection

Bruker SMART APEX CCD diffractometer
Absorption correction: multi-scan (*SADABS*; Bruker, 2000 ▶) *T*_min_ = 0.981, *T*_max_ = 0.985
13429 measured reflections
5463 independent reflections
3052 reflections with *I* > 2σ(*I*)
*R*_int_ = 0.039

#### Refinement

*R*[*F* ^2^ > 2σ(*F* ^2^)] = 0.048
*wR*(*F* ^2^) = 0.120
*S* = 1.06
5463 reflections
347 parameters
H-atom parameters constrained
Δρ_max_ = 0.16 e Å^−3^
Δρ_min_ = −0.18 e Å^−3^

### 

Data collection: *SMART* (Bruker, 2000 ▶); cell refinement: *SAINT* (Bruker, 2000 ▶); data reduction: *SAINT*; program(s) used to solve structure: *SHELXTL* (Sheldrick, 2008) ▶; program(s) used to refine structure: *SHELXTL*; molecular graphics: *SHELXTL*; software used to prepare material for publication: *SHELXTL*.

## Supplementary Material

Crystal structure: contains datablocks global, I. DOI: 10.1107/S1600536810047197/hg2745sup1.cif

Structure factors: contains datablocks I. DOI: 10.1107/S1600536810047197/hg2745Isup2.hkl

Additional supplementary materials:  crystallographic information; 3D view; checkCIF report

## Figures and Tables

**Fig. 1.**
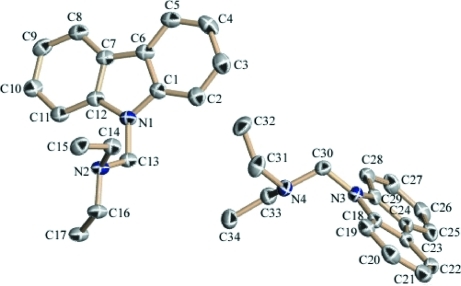
A view of the title compound showing the atom-numbering scheme and displacement ellipsoids drawn at 30% probability level. All H atoms have been omitted for clarity.

**Fig. 2.**
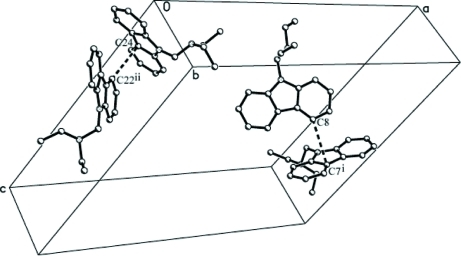
A view of the edge-to-face π–π stacking interactions. Dashed lines indicate weqk edge-to-face π–π stacking interactions and all H atoms have been omitted for clarity. (i: 1 - x,-1/2 + y,0.5 - z ii: -x,1/2 + y,0.5 - z)

**Fig. 3.**
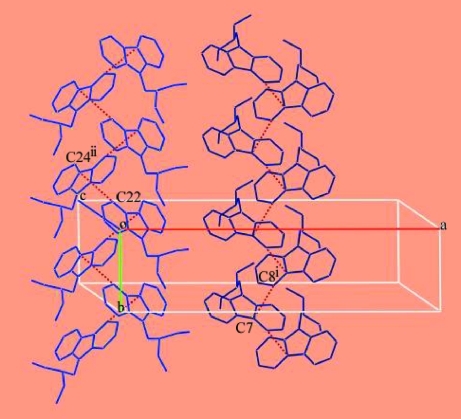
A view of the pillar structure. Dashed lines indicate weak edge-to-face π–π stacking interactions. All H atoms have been omitted for clarity. (i: 1 - x,-1/2 + y,0.5 - z ii: -x,1/2 + y,0.5 - z)

## supplementary crystallographic information

### Comment

Carbazole-based compounds are known for their intense luminescence and widely used in OLEDs as emitters (Adhikari *et al.*, 2007; Liu *et al.*, 2006; Palayangoda *et al.*, 2008). They can also be used as organic fluorescence probes (Hao *et al.*, 2010; Pappayee *et al.*, 2000). In our continuing studies in organic fluorescence probes (Shen *et al.*, 2008; Shen *et al.*, 2006), the 9-diethylaminomethyl carbazole(I) was synthesized.

The crystal structure of the title compound, C_17_H_20_N_2_, reveals that all the bond lengths and angles have normal values. Each asymmetric unit contains two title molecules, which are similar to each other with only slightly difference in their bond lengths and angles (Figure 1 and Table 1).

In the crystal packing the edge-to-face π–π stacking interactions were observed. The distance from the edge of the molecular plane B (N1^i^/C1^i^/C2^i^/C3^i^/C4^i^/C5^i^/C6^i^/C7^i^/C8^i^/C9^i^/ C10^i^/C11^i^/C12^i^)(i: 1 - *x*,-1/2 + *y*,0.5 - *z*) to the face of the molecular plane A(N1/C1/C2/C3/C4/C5/C6/C7/C8/C9/C10/C11/C12) is 3.538 (3) Å, and the dihedral angle between plane A and B is 62.06 (3)°. Similar relationships were observed with the molecular plane C and D. The edge-to-face distance of the molecular plane D(N3^ii^/C18^ii^/C19^ii^/C20^ii^/C21^ii^/C22^ii^/C23^ii^/ C24^ii^/C25^ii^/C26^ii^/C27^ii^/C28^ii^/C29^ii^)(ii: -*x*,1/2 + *y*,0.5 - *z*) to the molecular plane C(N3/C18/C19/C20/C21/C22/C23/C24/C25/C26/C27 /C28/C29) is 3.496 (3) Å, and the dihedral angle between plane C and D is 61.41 (3)° (Figure 2).Through these edge-to-face π–π stacking interactions, the neighbouring molecules form pillar structures (Figure 3).

### Experimental

9-Diethylaminomethyl carbazole was prepared according to a procedure described in the literature (Gu, *et al.*, 1997). Colorless crystals were obtained by recrystallized from ethanol at room temperature.

^1^H-NMR (CDCl_3_, 400 MHz) δ: 1.07 (t, 6H, 2-CH3), 2.68 (q, 4H, 2-CH2-), 4.98 (s, 2H, –CH2-), 7.22 (t, 2H, ArH), 7.44 (t, 2H, ArH), 7.54 (d, 2H, ArH), 8.06 (d, 2H, ArH).

### Refinement

The H atoms were placed in calculated positions and included as part of a riding model, with C—H = 0.93–0.97 Å, and with U_equiv_ values set at 1.2 U_equiv_ of the parent atoms.

### Crystal data

**Table:**

C_17_H_20_N_2_	*F*(000) = 1088
*M**_r_* = 252.35	*D*_x_ = 1.174 Mg m^−^^3^
Monoclinic, *P*2_1_/*c*	Mo *K*α radiation, λ = 0.71073 Å
Hall symbol: -P 2ybc	Cell parameters from 1470 reflections
*a* = 24.338 (2) Å	θ = 2.2–21.0°
*b* = 6.3216 (11) Å	µ = 0.07 mm^−^^1^
*c* = 19.133 (2) Å	*T* = 291 K
β = 104.109 (2)°	Block, colourless
*V* = 2854.9 (6) Å^3^	0.28 × 0.24 × 0.22 mm
*Z* = 8	

### Data collection

**Table:**

Bruker SMART APEX CCD diffractometer	5463 independent reflections
Radiation source: sealed tube	3052 reflections with *I* > 2σ(*I*)
graphite	*R*_int_ = 0.039
phi and ω scans	θ_max_ = 26.0°, θ_min_ = 0.9°
Absorption correction: multi-scan (*SADABS*; Bruker, 2000)	*h* = −25→29
*T*_min_ = 0.981, *T*_max_ = 0.985	*k* = −7→7
13429 measured reflections	*l* = −22→18

### Refinement

**Table:**

Refinement on *F*^2^	Primary atom site location: structure-invariant direct methods
Least-squares matrix: full	Secondary atom site location: difference Fourier map
*R*[*F*^2^ > 2σ(*F*^2^)] = 0.048	Hydrogen site location: inferred from neighbouring sites
*wR*(*F*^2^) = 0.120	H-atom parameters constrained
*S* = 1.06	*w* = 1/[σ^2^(*F*_o_^2^) + (0.05*P*)^2^] where *P* = (*F*_o_^2^ + 2*F*_c_^2^)/3
5463 reflections	(Δ/σ)_max_ < 0.001
347 parameters	Δρ_max_ = 0.16 e Å^−^^3^
0 restraints	Δρ_min_ = −0.18 e Å^−^^3^

### Special details

**Table:**

Geometry. All e.s.d.'s (except the e.s.d. in the dihedral angle between two l.s. planes) are estimated using the full covariance matrix. The cell e.s.d.'s are taken into account individually in the estimation of e.s.d.'s in distances, angles and torsion angles; correlations between e.s.d.'s in cell parameters are only used when they are defined by crystal symmetry. An approximate (isotropic) treatment of cell e.s.d.'s is used for estimating e.s.d.'s involving l.s. planes.Least-squares planes (*x*,*y*,*z* in crystal coordinates) and deviations from them (* indicates atom used to define plane)8.9285(0.0063)*x* - 3.2585(0.0028)*y* + 12.6587(0.0061)*z* = 2.4983(0.0046)* -0.0251 (0.0014) N1 * -0.0167 (0.0017) C1 * 0.0499 (0.0017) C2 * 0.0607 (0.0018) C3 * -0.0014 (0.0018) C4 * -0.0354 (0.0017) C5 * -0.0486 (0.0017) C6 * -0.0407 (0.0017) C7 * 0.0096 (0.0017) C8 * 0.0577 (0.0017) C9 * 0.0343 (0.0017) C10 * -0.0110 (0.0016) C11 * -0.0333 (0.0017) C12Rms deviation of fitted atoms = 0.03748.9285(0.0063)*x* + 3.2585(0.0028)*y* + 12.6588(0.0061)*z* = 11.1303(0.0032)Angle to previous plane (with approximate e.s.d.) = 62.06 (0.03)* 0.0251 (0.0014) N1_$1 * 0.0167 (0.0017) C1_$1 * -0.0499 (0.0017) C2_$1 * -0.0607 (0.0018) C3_$1 * 0.0014 (0.0018) C4_$1 * 0.0354 (0.0017) C5_$1 * 0.0486 (0.0017) C6_$1 * 0.0407 (0.0017) C7_$1 * -0.0096 (0.0017) C8_$1 * -0.0577 (0.0017) C9_$1 * -0.0343 (0.0017) C10_$1 * 0.0110 (0.0016) C11_$1 * 0.0333 (0.0017) C12_$1Rms deviation of fitted atoms = 0.0374-17.1148(0.0065)*x* + 3.2280(0.0027)*y* + 12.4596(0.0056)*z* = 1.3402(0.0013)Angle to previous plane (with approximate e.s.d.) = 67.15 (0.03)* -0.0095 (0.0014) N3 * -0.0154 (0.0016) C18 * -0.0081 (0.0017) C19 * 0.0048 (0.0018) C20 * 0.0356 (0.0017) C21 * 0.0196 (0.0016) C22 * -0.0218 (0.0017) C23 * -0.0287 (0.0017) C24 * -0.0272 (0.0016) C25 * -0.0051 (0.0017) C26 * 0.0333 (0.0018) C27 * 0.0347 (0.0016) C28 * -0.0123 (0.0016) C29Rms deviation of fitted atoms = 0.0225-17.1148(0.0065)*x* - 3.2280(0.0027)*y* + 12.4596(0.0056)*z* = 3.2755(0.0028)Angle to previous plane (with approximate e.s.d.) = 61.41 (0.03)* 0.0095 (0.0014) N3_$2 * 0.0154 (0.0016) C18_$2 * 0.0081 (0.0017) C19_$2 * -0.0048 (0.0018) C20_$2 * -0.0356 (0.0017) C21_$2 * -0.0196 (0.0016) C22_$2 * 0.0218 (0.0017) C23_$2 * 0.0287 (0.0017) C24_$2 * 0.0272 (0.0016) C25_$2 * 0.0051 (0.0017) C26_$2 * -0.0333 (0.0018) C27_$2 * -0.0347 (0.0016) C28_$2 * 0.0123 (0.0016) C29_$2Rms deviation of fitted atoms = 0.0225
Refinement. Refinement of *F*^2^ against ALL reflections. The weighted *R*-factor *wR* and goodness of fit *S* are based on *F*^2^, conventional *R*-factors *R* are based on *F*, with *F* set to zero for negative *F*^2^. The threshold expression of *F*^2^ > σ(*F*^2^) is used only for calculating *R*-factors(gt) *etc*. and is not relevant to the choice of reflections for refinement. *R*-factors based on *F*^2^ are statistically about twice as large as those based on *F*, and *R*- factors based on ALL data will be even larger.

### Fractional atomic coordinates and isotropic or equivalent isotropic displacement parameters (Å^2^)

**Table:**

	*x*	*y*	*z*	*U*_iso_*/*U*_eq_	
C1	0.36426 (8)	0.9208 (3)	0.17614 (10)	0.0376 (4)	
C2	0.31632 (9)	0.8391 (4)	0.19419 (11)	0.0458 (5)	
H2	0.3006	0.7112	0.1752	0.055*	
C3	0.29289 (10)	0.9544 (4)	0.24125 (11)	0.0539 (6)	
H3	0.2610	0.9027	0.2543	0.065*	
C4	0.31618 (11)	1.1462 (4)	0.26928 (12)	0.0580 (6)	
H4	0.2990	1.2226	0.2996	0.070*	
C5	0.36412 (10)	1.2249 (3)	0.25303 (10)	0.0469 (5)	
H5	0.3799	1.3513	0.2733	0.056*	
C6	0.38897 (8)	1.1136 (3)	0.20581 (10)	0.0395 (5)	
C7	0.43876 (9)	1.1426 (3)	0.17880 (10)	0.0380 (5)	
C8	0.48144 (10)	1.2920 (3)	0.19114 (11)	0.0440 (5)	
H8	0.4804	1.4063	0.2214	0.053*	
C9	0.52563 (9)	1.2705 (3)	0.15822 (12)	0.0489 (6)	
H9	0.5547	1.3698	0.1671	0.059*	
C10	0.52730 (9)	1.1005 (3)	0.11143 (12)	0.0471 (5)	
H10	0.5570	1.0900	0.0889	0.057*	
C11	0.48519 (9)	0.9490 (3)	0.09856 (10)	0.0413 (5)	
H11	0.4860	0.8360	0.0677	0.050*	
C12	0.44188 (8)	0.9704 (3)	0.13283 (10)	0.0363 (4)	
C13	0.38250 (9)	0.6526 (3)	0.08628 (10)	0.0390 (5)	
H13A	0.3519	0.5759	0.0993	0.047*	
H13B	0.4153	0.5601	0.0946	0.047*	
C14	0.31317 (9)	0.8340 (3)	−0.00586 (11)	0.0422 (5)	
H14A	0.3163	0.9432	0.0305	0.051*	
H14B	0.2817	0.7431	−0.0028	0.051*	
C15	0.30047 (10)	0.9368 (3)	−0.07941 (11)	0.0494 (5)	
H15A	0.3329	1.0161	−0.0846	0.074*	
H15B	0.2686	1.0299	−0.0844	0.074*	
H15C	0.2919	0.8295	−0.1160	0.074*	
C16	0.35944 (9)	0.5149 (3)	−0.03411 (11)	0.0446 (5)	
H16A	0.3432	0.5523	−0.0841	0.054*	
H16B	0.3331	0.4205	−0.0190	0.054*	
C17	0.41427 (9)	0.3987 (3)	−0.02917 (12)	0.0468 (5)	
H17A	0.4436	0.4985	−0.0311	0.070*	
H17B	0.4098	0.3016	−0.0687	0.070*	
H17C	0.4244	0.3220	0.0154	0.070*	
C18	0.04584 (8)	0.0516 (3)	0.15594 (10)	0.0351 (4)	
C19	0.00476 (9)	0.0810 (4)	0.09248 (11)	0.0446 (5)	
H19	0.0056	0.1976	0.0631	0.053*	
C20	−0.03736 (9)	−0.0685 (4)	0.07439 (12)	0.0520 (6)	
H20	−0.0650	−0.0531	0.0315	0.062*	
C21	−0.04000 (9)	−0.2432 (4)	0.11849 (12)	0.0522 (6)	
H21	−0.0696	−0.3397	0.1053	0.063*	
C22	0.00128 (8)	−0.2722 (3)	0.18142 (11)	0.0436 (5)	
H22	−0.0001	−0.3881	0.2109	0.052*	
C23	0.04508 (8)	−0.1255 (3)	0.20027 (10)	0.0364 (4)	
C24	0.09355 (8)	−0.1064 (3)	0.26133 (10)	0.0365 (4)	
C25	0.11486 (9)	−0.2287 (4)	0.32240 (11)	0.0446 (5)	
H25	0.0971	−0.3544	0.3295	0.054*	
C26	0.16223 (9)	−0.1619 (4)	0.37195 (11)	0.0503 (6)	
H26	0.1768	−0.2443	0.4125	0.060*	
C27	0.18903 (9)	0.0279 (4)	0.36267 (11)	0.0494 (5)	
H27	0.2209	0.0708	0.3973	0.059*	
C28	0.16878 (9)	0.1523 (3)	0.30273 (11)	0.0431 (5)	
H28	0.1865	0.2791	0.2968	0.052*	
C29	0.12153 (8)	0.0847 (3)	0.25158 (10)	0.0343 (4)	
C30	0.10458 (9)	0.3792 (3)	0.15881 (11)	0.0409 (5)	
H30A	0.1289	0.4608	0.1971	0.049*	
H30B	0.0694	0.4569	0.1422	0.049*	
C31	0.12229 (10)	0.5486 (4)	0.05375 (12)	0.0543 (6)	
H31A	0.0819	0.5757	0.0397	0.065*	
H31B	0.1345	0.5186	0.0101	0.065*	
C32	0.15171 (11)	0.7484 (3)	0.08706 (14)	0.0623 (7)	
H32A	0.1410	0.7785	0.1311	0.093*	
H32B	0.1408	0.8643	0.0542	0.093*	
H32C	0.1920	0.7292	0.0970	0.093*	
C33	0.19210 (9)	0.3007 (3)	0.12612 (12)	0.0464 (5)	
H33A	0.1946	0.1725	0.1546	0.056*	
H33B	0.2113	0.4122	0.1576	0.056*	
C34	0.22266 (11)	0.2653 (4)	0.06688 (14)	0.0658 (7)	
H34A	0.2016	0.1667	0.0324	0.099*	
H34B	0.2598	0.2094	0.0874	0.099*	
H34C	0.2259	0.3972	0.0434	0.099*	
N1	0.39612 (7)	0.8375 (2)	0.13156 (8)	0.0365 (4)	
N2	0.36559 (6)	0.7080 (2)	0.00993 (8)	0.0359 (4)	
N3	0.09245 (7)	0.1786 (2)	0.18752 (8)	0.0353 (4)	
N4	0.13199 (7)	0.3584 (2)	0.09955 (9)	0.0391 (4)	

### Atomic displacement parameters (Å^2^)

**Table:**

	*U*^11^	*U*^22^	*U*^33^	*U*^12^	*U*^13^	*U*^23^
C1	0.0431 (11)	0.0382 (10)	0.0286 (9)	0.0088 (9)	0.0031 (8)	0.0040 (8)
C2	0.0447 (12)	0.0515 (13)	0.0404 (11)	0.0040 (10)	0.0085 (10)	0.0047 (10)
C3	0.0502 (13)	0.0698 (16)	0.0412 (12)	0.0061 (12)	0.0100 (11)	0.0061 (11)
C4	0.0629 (15)	0.0736 (17)	0.0377 (12)	0.0200 (14)	0.0129 (11)	−0.0067 (12)
C5	0.0649 (15)	0.0367 (11)	0.0328 (10)	0.0090 (10)	−0.0003 (11)	−0.0009 (9)
C6	0.0433 (11)	0.0376 (11)	0.0311 (9)	0.0080 (9)	−0.0037 (9)	0.0007 (8)
C7	0.0425 (11)	0.0332 (10)	0.0303 (10)	0.0068 (9)	−0.0062 (8)	0.0003 (8)
C8	0.0568 (13)	0.0342 (10)	0.0335 (10)	0.0041 (10)	−0.0032 (10)	−0.0009 (8)
C9	0.0454 (12)	0.0396 (11)	0.0527 (13)	−0.0095 (10)	−0.0056 (11)	0.0034 (10)
C10	0.0382 (11)	0.0453 (12)	0.0540 (13)	−0.0005 (10)	0.0036 (10)	−0.0018 (10)
C11	0.0440 (11)	0.0342 (11)	0.0423 (11)	0.0037 (9)	0.0038 (10)	−0.0023 (9)
C12	0.0346 (10)	0.0307 (10)	0.0384 (10)	0.0082 (8)	−0.0011 (8)	0.0021 (8)
C13	0.0434 (11)	0.0300 (9)	0.0415 (11)	0.0006 (9)	0.0059 (9)	−0.0054 (9)
C14	0.0420 (11)	0.0409 (11)	0.0405 (11)	0.0065 (9)	0.0039 (10)	0.0005 (9)
C15	0.0531 (13)	0.0404 (11)	0.0490 (12)	−0.0012 (10)	0.0015 (10)	0.0074 (9)
C16	0.0457 (12)	0.0346 (10)	0.0504 (11)	0.0004 (9)	0.0057 (10)	−0.0151 (9)
C17	0.0482 (13)	0.0360 (11)	0.0533 (13)	0.0020 (9)	0.0071 (10)	−0.0125 (9)
C18	0.0368 (10)	0.0385 (10)	0.0329 (9)	0.0014 (8)	0.0144 (9)	−0.0014 (8)
C19	0.0453 (12)	0.0541 (12)	0.0358 (10)	0.0013 (11)	0.0126 (9)	−0.0022 (9)
C20	0.0386 (12)	0.0752 (16)	0.0411 (11)	0.0002 (11)	0.0074 (10)	−0.0129 (11)
C21	0.0415 (12)	0.0636 (15)	0.0562 (13)	−0.0151 (11)	0.0209 (11)	−0.0211 (12)
C22	0.0447 (12)	0.0429 (11)	0.0499 (12)	−0.0078 (10)	0.0246 (11)	−0.0083 (9)
C23	0.0372 (10)	0.0412 (11)	0.0350 (10)	0.0002 (9)	0.0168 (9)	−0.0025 (9)
C24	0.0407 (11)	0.0371 (10)	0.0360 (10)	0.0031 (9)	0.0175 (9)	−0.0021 (9)
C25	0.0480 (13)	0.0478 (12)	0.0436 (11)	0.0107 (10)	0.0216 (11)	0.0070 (10)
C26	0.0487 (13)	0.0693 (15)	0.0370 (12)	0.0180 (12)	0.0180 (10)	0.0081 (10)
C27	0.0374 (11)	0.0752 (16)	0.0353 (10)	0.0007 (11)	0.0080 (9)	−0.0005 (11)
C28	0.0456 (12)	0.0472 (12)	0.0405 (11)	−0.0020 (10)	0.0180 (10)	−0.0077 (9)
C29	0.0363 (10)	0.0382 (10)	0.0314 (9)	0.0026 (8)	0.0139 (8)	0.0008 (8)
C30	0.0459 (11)	0.0278 (10)	0.0530 (12)	0.0014 (9)	0.0194 (10)	−0.0005 (9)
C31	0.0524 (13)	0.0558 (14)	0.0514 (13)	−0.0038 (11)	0.0060 (11)	0.0236 (11)
C32	0.0694 (17)	0.0346 (12)	0.0866 (18)	0.0006 (11)	0.0263 (14)	0.0193 (12)
C33	0.0524 (13)	0.0381 (11)	0.0531 (13)	0.0045 (10)	0.0214 (11)	0.0052 (9)
C34	0.0804 (19)	0.0502 (14)	0.0831 (18)	0.0030 (13)	0.0516 (16)	0.0091 (13)
N1	0.0406 (9)	0.0327 (8)	0.0347 (9)	0.0007 (7)	0.0065 (7)	−0.0038 (7)
N2	0.0356 (8)	0.0309 (8)	0.0367 (9)	0.0040 (7)	0.0003 (7)	−0.0064 (7)
N3	0.0428 (9)	0.0309 (8)	0.0344 (8)	−0.0010 (7)	0.0135 (7)	0.0030 (6)
N4	0.0481 (10)	0.0327 (9)	0.0389 (9)	0.0014 (7)	0.0151 (8)	0.0065 (7)

### Geometric parameters (Å, °)

**Table:**

C1—N1	1.389 (2)	C18—C19	1.384 (3)
C1—C2	1.394 (3)	C18—N3	1.401 (2)
C1—C6	1.415 (3)	C18—C23	1.408 (3)
C2—C3	1.385 (3)	C19—C20	1.375 (3)
C2—H2	0.9300	C19—H19	0.9300
C3—C4	1.389 (3)	C20—C21	1.401 (3)
C3—H3	0.9300	C20—H20	0.9300
C4—C5	1.372 (3)	C21—C22	1.380 (3)
C4—H4	0.9300	C21—H21	0.9300
C5—C6	1.394 (3)	C22—C23	1.392 (3)
C5—H5	0.9300	C22—H22	0.9300
C6—C7	1.440 (3)	C23—C24	1.449 (3)
C7—C8	1.381 (3)	C24—C25	1.392 (3)
C7—C12	1.413 (3)	C24—C29	1.421 (3)
C8—C9	1.379 (3)	C25—C26	1.369 (3)
C8—H8	0.9300	C25—H25	0.9300
C9—C10	1.406 (3)	C26—C27	1.398 (3)
C9—H9	0.9300	C26—H26	0.9300
C10—C11	1.380 (3)	C27—C28	1.379 (3)
C10—H10	0.9300	C27—H27	0.9300
C11—C12	1.377 (3)	C28—C29	1.384 (3)
C11—H11	0.9300	C28—H28	0.9300
C12—N1	1.390 (2)	C29—N3	1.390 (2)
C13—N1	1.445 (2)	C30—N3	1.440 (2)
C13—N2	1.460 (2)	C30—N4	1.455 (2)
C13—H13A	0.9700	C30—H30A	0.9700
C13—H13B	0.9700	C30—H30B	0.9700
C14—N2	1.471 (2)	C31—N4	1.473 (3)
C14—C15	1.512 (3)	C31—C32	1.514 (3)
C14—H14A	0.9700	C31—H31A	0.9700
C14—H14B	0.9700	C31—H31B	0.9700
C15—H15A	0.9600	C32—H32A	0.9600
C15—H15B	0.9600	C32—H32B	0.9600
C15—H15C	0.9600	C32—H32C	0.9600
C16—N2	1.470 (2)	C33—N4	1.473 (3)
C16—C17	1.506 (3)	C33—C34	1.516 (3)
C16—H16A	0.9700	C33—H33A	0.9700
C16—H16B	0.9700	C33—H33B	0.9700
C17—H17A	0.9600	C34—H34A	0.9600
C17—H17B	0.9600	C34—H34B	0.9600
C17—H17C	0.9600	C34—H34C	0.9600
			
N1—C1—C2	129.63 (19)	C19—C20—C21	122.1 (2)
N1—C1—C6	109.08 (18)	C19—C20—H20	119.0
C2—C1—C6	121.27 (19)	C21—C20—H20	119.0
C3—C2—C1	117.9 (2)	C22—C21—C20	120.0 (2)
C3—C2—H2	121.0	C22—C21—H21	120.0
C1—C2—H2	121.0	C20—C21—H21	120.0
C2—C3—C4	121.1 (2)	C21—C22—C23	119.1 (2)
C2—C3—H3	119.5	C21—C22—H22	120.4
C4—C3—H3	119.5	C23—C22—H22	120.4
C5—C4—C3	121.2 (2)	C22—C23—C18	119.69 (18)
C5—C4—H4	119.4	C22—C23—C24	133.32 (19)
C3—C4—H4	119.4	C18—C23—C24	106.96 (17)
C4—C5—C6	119.4 (2)	C25—C24—C29	119.35 (19)
C4—C5—H5	120.3	C25—C24—C23	133.94 (19)
C6—C5—H5	120.3	C29—C24—C23	106.69 (17)
C5—C6—C1	119.1 (2)	C26—C25—C24	119.3 (2)
C5—C6—C7	134.7 (2)	C26—C25—H25	120.4
C1—C6—C7	106.15 (17)	C24—C25—H25	120.4
C8—C7—C12	118.8 (2)	C25—C26—C27	121.1 (2)
C8—C7—C6	133.57 (19)	C25—C26—H26	119.4
C12—C7—C6	107.61 (17)	C27—C26—H26	119.4
C9—C8—C7	119.48 (19)	C28—C27—C26	120.8 (2)
C9—C8—H8	120.3	C28—C27—H27	119.6
C7—C8—H8	120.3	C26—C27—H27	119.6
C8—C9—C10	120.9 (2)	C27—C28—C29	118.6 (2)
C8—C9—H9	119.6	C27—C28—H28	120.7
C10—C9—H9	119.6	C29—C28—H28	120.7
C11—C10—C9	120.6 (2)	C28—C29—N3	130.39 (18)
C11—C10—H10	119.7	C28—C29—C24	120.81 (18)
C9—C10—H10	119.7	N3—C29—C24	108.77 (16)
C12—C11—C10	117.93 (19)	N3—C30—N4	113.15 (15)
C12—C11—H11	121.0	N3—C30—H30A	108.9
C10—C11—H11	121.0	N4—C30—H30A	108.9
C11—C12—N1	129.44 (17)	N3—C30—H30B	108.9
C11—C12—C7	122.36 (19)	N4—C30—H30B	108.9
N1—C12—C7	108.18 (17)	H30A—C30—H30B	107.8
N1—C13—N2	111.93 (15)	N4—C31—C32	116.10 (18)
N1—C13—H13A	109.2	N4—C31—H31A	108.3
N2—C13—H13A	109.2	C32—C31—H31A	108.3
N1—C13—H13B	109.2	N4—C31—H31B	108.3
N2—C13—H13B	109.2	C32—C31—H31B	108.3
H13A—C13—H13B	107.9	H31A—C31—H31B	107.4
N2—C14—C15	113.21 (17)	C31—C32—H32A	109.5
N2—C14—H14A	108.9	C31—C32—H32B	109.5
C15—C14—H14A	108.9	H32A—C32—H32B	109.5
N2—C14—H14B	108.9	C31—C32—H32C	109.5
C15—C14—H14B	108.9	H32A—C32—H32C	109.5
H14A—C14—H14B	107.7	H32B—C32—H32C	109.5
C14—C15—H15A	109.5	N4—C33—C34	113.93 (19)
C14—C15—H15B	109.5	N4—C33—H33A	108.8
H15A—C15—H15B	109.5	C34—C33—H33A	108.8
C14—C15—H15C	109.5	N4—C33—H33B	108.8
H15A—C15—H15C	109.5	C34—C33—H33B	108.8
H15B—C15—H15C	109.5	H33A—C33—H33B	107.7
N2—C16—C17	113.83 (16)	C33—C34—H34A	109.5
N2—C16—H16A	108.8	C33—C34—H34B	109.5
C17—C16—H16A	108.8	H34A—C34—H34B	109.5
N2—C16—H16B	108.8	C33—C34—H34C	109.5
C17—C16—H16B	108.8	H34A—C34—H34C	109.5
H16A—C16—H16B	107.7	H34B—C34—H34C	109.5
C16—C17—H17A	109.5	C1—N1—C12	108.96 (15)
C16—C17—H17B	109.5	C1—N1—C13	126.47 (17)
H17A—C17—H17B	109.5	C12—N1—C13	124.41 (16)
C16—C17—H17C	109.5	C13—N2—C16	109.79 (15)
H17A—C17—H17C	109.5	C13—N2—C14	110.73 (15)
H17B—C17—H17C	109.5	C16—N2—C14	111.71 (15)
C19—C18—N3	129.58 (18)	C29—N3—C18	108.65 (15)
C19—C18—C23	121.49 (18)	C29—N3—C30	127.21 (17)
N3—C18—C23	108.92 (16)	C18—N3—C30	124.03 (16)
C20—C19—C18	117.6 (2)	C30—N4—C33	111.05 (16)
C20—C19—H19	121.2	C30—N4—C31	110.64 (16)
C18—C19—H19	121.2	C33—N4—C31	114.16 (17)
			
N1—C1—C2—C3	179.13 (19)	C23—C24—C25—C26	−178.5 (2)
C6—C1—C2—C3	1.1 (3)	C24—C25—C26—C27	0.8 (3)
C1—C2—C3—C4	0.4 (3)	C25—C26—C27—C28	−0.7 (3)
C2—C3—C4—C5	−2.0 (3)	C26—C27—C28—C29	−0.4 (3)
C3—C4—C5—C6	1.9 (3)	C27—C28—C29—N3	179.32 (19)
C4—C5—C6—C1	−0.4 (3)	C27—C28—C29—C24	1.5 (3)
C4—C5—C6—C7	−177.7 (2)	C25—C24—C29—C28	−1.4 (3)
N1—C1—C6—C5	−179.48 (17)	C23—C24—C29—C28	177.65 (17)
C2—C1—C6—C5	−1.1 (3)	C25—C24—C29—N3	−179.67 (16)
N1—C1—C6—C7	−1.5 (2)	C23—C24—C29—N3	−0.6 (2)
C2—C1—C6—C7	176.88 (18)	C2—C1—N1—C12	−176.97 (19)
C5—C6—C7—C8	1.5 (4)	C6—C1—N1—C12	1.2 (2)
C1—C6—C7—C8	−176.0 (2)	C2—C1—N1—C13	7.6 (3)
C5—C6—C7—C12	178.7 (2)	C6—C1—N1—C13	−174.21 (16)
C1—C6—C7—C12	1.2 (2)	C11—C12—N1—C1	178.27 (19)
C12—C7—C8—C9	0.4 (3)	C7—C12—N1—C1	−0.5 (2)
C6—C7—C8—C9	177.3 (2)	C11—C12—N1—C13	−6.2 (3)
C7—C8—C9—C10	1.0 (3)	C7—C12—N1—C13	175.11 (16)
C8—C9—C10—C11	−1.3 (3)	N2—C13—N1—C1	108.6 (2)
C9—C10—C11—C12	0.1 (3)	N2—C13—N1—C12	−66.2 (2)
C10—C11—C12—N1	−177.16 (18)	N1—C13—N2—C16	172.62 (17)
C10—C11—C12—C7	1.4 (3)	N1—C13—N2—C14	−63.6 (2)
C8—C7—C12—C11	−1.6 (3)	C17—C16—N2—C13	−66.3 (2)
C6—C7—C12—C11	−179.32 (17)	C17—C16—N2—C14	170.45 (18)
C8—C7—C12—N1	177.19 (17)	C15—C14—N2—C13	167.25 (16)
C6—C7—C12—N1	−0.5 (2)	C15—C14—N2—C16	−70.0 (2)
N3—C18—C19—C20	−178.99 (19)	C28—C29—N3—C18	−177.64 (19)
C23—C18—C19—C20	0.6 (3)	C24—C29—N3—C18	0.4 (2)
C18—C19—C20—C21	1.2 (3)	C28—C29—N3—C30	−1.4 (3)
C19—C20—C21—C22	−1.6 (3)	C24—C29—N3—C30	176.64 (16)
C20—C21—C22—C23	0.2 (3)	C19—C18—N3—C29	179.61 (19)
C21—C22—C23—C18	1.5 (3)	C23—C18—N3—C29	0.0 (2)
C21—C22—C23—C24	179.0 (2)	C19—C18—N3—C30	3.2 (3)
C19—C18—C23—C22	−1.9 (3)	C23—C18—N3—C30	−176.41 (16)
N3—C18—C23—C22	177.72 (16)	N4—C30—N3—C29	104.2 (2)
C19—C18—C23—C24	179.99 (17)	N4—C30—N3—C18	−80.1 (2)
N3—C18—C23—C24	−0.4 (2)	N3—C30—N4—C33	−75.7 (2)
C22—C23—C24—C25	1.7 (4)	N3—C30—N4—C31	156.47 (18)
C18—C23—C24—C25	179.5 (2)	C34—C33—N4—C30	177.03 (18)
C22—C23—C24—C29	−177.1 (2)	C34—C33—N4—C31	−57.1 (2)
C18—C23—C24—C29	0.6 (2)	C32—C31—N4—C30	69.2 (3)
C29—C24—C25—C26	0.2 (3)	C32—C31—N4—C33	−56.9 (3)
